# RSK2 promotes melanoma cell proliferation and vemurafenib resistance *via* upregulating cyclin D1

**DOI:** 10.3389/fphar.2022.950571

**Published:** 2022-09-20

**Authors:** Hai-Zhou Wu, Lan-Ya Li, Shi-Long Jiang, Yi-Zhi Li, Xiao-Mei Shi, Xin-Yuan Sun, Zhuo Li, Yan Cheng

**Affiliations:** ^1^ Department of Pharmacy, The Second Xiangya Hospital, Central South University, Changsha, China; ^2^ Xiangya School of Pharmaceutical Sciences, Central South University, Changsha, China; ^3^ Hunan Provincial Engineering Research Centre of Translational Medicine and Innovative Drug, Changsha, China; ^4^ Department of ophthalmology, The Second Xiangya Hospital, Central South University, Changsha, China

**Keywords:** melanoma, Rsk2, vemurafenib, cyclin D1, FoxO1

## Abstract

BRAF inhibitors are commonly used in targeted therapies for melanoma patients harboring BRAF^V600E^ mutant. Despite the benefit of vemurafenib therapy, acquired resistance during or after treatment remains a major obstacle in BRAF^V600E^ mutant melanoma. Here we found that RSK2 is overexpressed in melanoma cells and the high expression of RSK2 indicates poor overall survival (OS) in melanoma patients. Overexpression of RSK2 leads to vemurafenib resistance, and the deletion of RSK2 inhibits cell proliferation and sensitizes melanoma cells to vemurafenib. Mechanistically, RSK2 enhances the phosphorylation of FOXO1 by interacting with FOXO1 and promoting its subsequent degradation, leading to upregulation of cyclin D1 in melanoma cells. These results not only reveal the presence of a RSK2-FOXO1-cyclin D1 signaling pathway in melanoma, but also provide a potential therapeutic strategy to enhance the efficacy of vemurafenib against cancer.

## Introduction

Melanoma is an aggressive skin cancer with high mortality, accounting for ∼100,530 new cases in 2020 (Siegel et al., 2020). Mutations at codon 600 of BRAF gene is widespread in melanoma (mostly V600E), making it a potential therapeutic target (Holderfield et al., 2014). As a BRAF inhibitor, vemurafenib shows great clinical benefit and has been approved by the United States Food and Drug Administration (FDA) as the first-line treatment for BRAF-mutated melanoma (Kramkimel et al., 2016). However, most patients treated with vemurafenib develop resistance (acquired resistance) after a relatively short period of disease control. Vemurafenib resistance is currently a persistent clinical problem in the management of BRAF mutant melanoma. Thus, it is an urgent problem to find the mechanism of vemurafenib resistance and effective sensitizers to prolong the survival rate of patients with malignant melanoma.

p90 ribosomal S6 kinase 2 (RSK2), belonging to the RSKs serine/threonine kinase family, is a downstream effector of the MAPK signaling cascade (Sulzmaier and Ramos, 2013). RSK2 is involved in various cellular processes, such as gene expression, cell cycle, motility, proliferation and apoptosis in various cancers by phosphorylating multiple signaling effectors (Kang and Chen, 2011). RSK2 is highly expressed in many types of cancers and promotes tumor growth and survival (Clark et al., 2005; Cho et al., 2012; Huynh et al., 2020). We previously found that RSK2 promoted autophagy under endoplasmic reticulum stress *via* phosphorylating AMPKα2 and inhibition of RSK2 enhanced the sensitivity of breast cancer cells to paclitaxel (Li et al., 2020). Furthermore, RSK2 is also associated with cisplatin resistance and lenalidomide resistance (van Jaarsveld et al., 2013; Zhu et al., 2015). Recent studies indicated that targeting RSK2 could suppress cutaneous melanoma cell proliferation and metastasis, alleviating the BRAF^V600E^ inhibitor resistance (Kosnopfel et al., 2017; Zhang et al., 2019). However, the mechanism regulated by RSK2 in vemurafenib-resistant melanoma cells remains obscure.

Cyclin D1, a critical cell cycle regulator encoded by the CCND1 gene, partners with CDK4/6 to promote cell cycle progression through driving transition from G1 to S phase (Qie and Diehl, 2016; Gonzalez-Ruiz et al., 2020). CCND1 is defined as an oncogene amplified in several tumors including melanoma (Casimiro et al., 2014). Overexpression of cyclin D1 has also been found in up to 62% of primary melanomas compared with melanocytic naevi (Kaufmann et al., 2020). The main oncogenic effect of cyclin D1/CCND1 upregulation or gene amplification is to promote tumor cell proliferation (Witzel et al., 2010). Previous studies showed that cyclin D1 overexpression might be sufficient to render BRAF^V600E^ melanoma cells resistant to vemurafenib (Yadav et al., 2015).

FOXO1, a multifunctional transcription factor, is one of the key substrates of PI3K/AKT signaling pathway and acts as a pivotal regulators of cell cycle progression (Wu et al., 2019). Active AKT increased the expression of cyclin D1 through promoting the phosphorylation and the degradation of FOXO1 (Cai et al., 2018; Li et al., 2019; Wu et al., 2019). Increasing evidence indicates that inhibition of FOXO1 in cancer cells promoted cell cycle transition and cell proliferation by upregulation of cyclin D1 (Sulzmaier and Ramos, 2013; Zhou et al., 2019). Thus, FOXO1/cyclin D1 signaling plays a central role in cell cycle progression and cell proliferation.

In this study, we investigated the biological role and the mechanism of RSK2 in the regulation of melanoma proliferation and vemurafenib resistance. We found that RSK2 promoted melanoma cell proliferation and vemurafenib resistance by upregulating cyclin D1 expression. Meanwhile, we also uncovered that RSK2-mediated cyclin D1 upregulation is facilitated by promoting FOXO1 degradation. Therefore, this study suggested that RSK2 was involved in acquired resistance of vemurafenib, and targeting RSK2 might be an effective way to increase the therapeutic effect of vemurafenib.

## Materials and methods

### Cell lines and culture

The human melanoma cell line A375 was purchased from Cell Bank of Chinese Academy of Sciences. Vemurafenib-resistant A375 cells (A375-VR) were established in our laboratory. A375 cells were cultured in DMEM/High glucose medium with 10% FBS and 1% Penicillin/Streptomycin solution. Vemurafenib-resistant A375-VR cells were maintained in DMEM/High with 10% FBS, 1% Penicillin/Streptomycin and 1.0 μM vemurafenib. All the cells above were grown at 37°C with 5% CO_2_.

### Reagents and antibodies

Vemurafenib and MG132 were purchased from Selleck Chemicals. Cycloheximide (CHX) was purchased from Amresco. Antibodies used in immunoblotting were purchased from Cell Signaling Technologies: RSK2 (No.5528, 1:1000), FOXO1 (No.2880,1:1000), p-FOXO1/Ser319(No.2486,1:1000) and cyclin D1 (No.55506, 1:1000). Anti-β-actin (No.60008-1-Ig,1:5000) was purchased from Proteintech.

### Cell viability assay

Cells were plated at 5 × 10^3^ cells per well in 96-well plates and were then treated with a series concentration of vemurafenib. After the treatment, cell viability was measured by Cell Counting Kit-8 assay following the manufacturer’s protocol (Selleck).

### EdU assay

Cells were incubated with 50 μM 5-Ethynyl-2′-deoxyuridine assay (EdU, RiboBio) for 2 h at 37°C. 4% paraformaldehyde was used to fix the cells for 30 min. After cells were treated with 2 mg/ml glycine for 5 min and incubated with 0.5% Triton X-100 for 10min. Then, cells were stained with 1× Apollo reaction cocktail for 30 min and exposed to Hoechst 33342 for 30 min at room temperature. Images were captured under a fluorescent microscope.

### Clonogenicity assay

The cells were plated at 800 cells per well in six-well culture plate for 10 days. After colony formation, the cells were washed with PBS and immobilized by 4% formaldehyde at room temperature for 30 min. Finally, the cells were dyed with 0.5% crystal violet for 30 min.

### RNA isolation and RT-qPCR

Total RNA was isolated using Trizol reagent (Cwbiotech) according to the manufacturer’s instruction and was reverse-transcribed by using PrimeScript RT Reagent Kit (Perfect real time) (Takara). Real time PCR was performed using iTap universal SYBR Green (Bio-rad), and was run on CFX96 system (Bio-Rad). For quantification of gene expression, the 2^−ΔΔCt^ method was used. GAPDH expression was used for normalization. The qPCR primer sets: cyclin D1:5′-TGCATCTACACCGACAACTCC-3′ (forward) and 5′-CGT​GTT​TGC​GGA​TGA​TCT​GTT-3′ (reverse), GAPDH: 5′-ACC​ACA​GTC​CAT​GCC​ATC​AC-3′ (forward) and 5′-TCC​ACC​ACC​CTG​TTG​CTG​TA-3′ (reverse), FOXO1:forward 5’ -CTT​CAA​GGA​TAA​GGG​CGA​CA-3′ (forward) and 5′ -ATT​TAA​GCG​GTG​TTA​GAC​AG-3′ (reverse).

### Immunoprecipitation assay

Cells were lysed with RIPA buffer with a protease inhibitor cocktail (Selleck). The indicated primary antibody was added to cell lysates containing protein A/G agarose beads (Santa Cruz, SC-2003) at 4°C overnight. The beads were washed four times with the cooled RIPA buffer, and the proteins were eluted by SDS-PAGE sample loading buffer and analyzed by immunoblotting.

### Western blot analysis

Cells were lysed with RIPA buffer with a protease inhibitor cocktail (Selleck) following by centrifugation at 14,000 ×g for 15 min. Protein concentrations of the lysates were determined by BCA assay kit. Equivalent amounts of cellular protein (15–30 μg) were separated by SDS-PAGE and transferred to PVDF membranes. And then, the membranes were blocked with 5% skim milk in PBST. The membranes were incubated with primary antibodies overnight and then peroxidase-conjugated secondary antibodies at room temperature for 1 h. Finally, the membranes were visualized with an enhanced chemiluminescent detection kit.

### Bioinformatics analysis

GPEIA2 tool were used to explore RSK2 expression between melanoma tissues and normal tissues and in different subtypes of melanoma. The Human Protein Atlas database was used to explore the protein level of RSK2 in human melanoma and normal tissues. The clinicopathological data of melanoma patients were obtained from TCGA using UCSC Xena. GSE46517, GSE22155, GSE99923, GSE15605 and GSE77940 were downloaded from the GEO database. GSE46517 was used to examine gene expression in normal tissues and melanoma tissues. The survival probability was evaluated with the Kaplan-Meier method, and the differences were determined by the log-rank test. GSE99923 was used to examine gene expression in vemurafenib sensitive and resistant cells. The gene set enrichment analysis (GSEA) was performed with GSEA-4.0 software (http://software.broad institute.org/gsea/). The correlation coefficients of RSK2 and CCND1 were determined by Spearman’s correlation test. The data of RSK2 mRNA expression and sensitivity to compounds were downloaded from cell minner (https://discover.nci.nih.gov/cellminer/home.do) and converted to Z-scores. The association between RSK2 and the efficacy of different compounds were tested by Pearson’s-correlation analysis.

### Statistical analysis

The difference between the samples with or without silencing of RSK2 expression was analyzed using unpaired two-tailed Student’s t-test. All experiments were performed at least three times. A value of *p* < 0.05 was considered to be significant. Statistical significance is displayed as *p < 0.05, **p < 0.01.

## Results

### RSK2 is highly expressed and is associated with poor prognosis in melanoma

**FIGURE 1:**
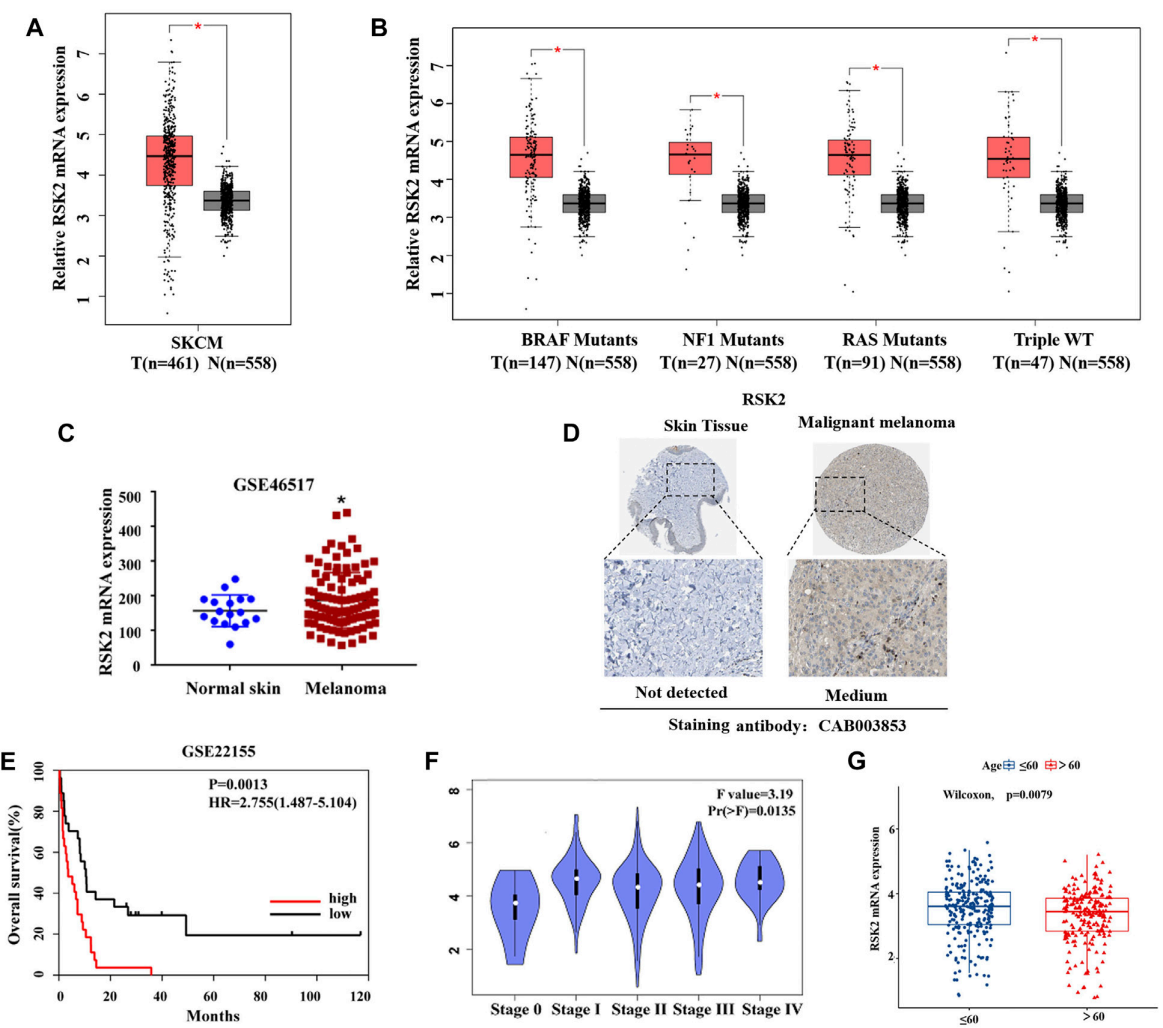
RSK2 is upregulated and is associated with poor prognosis in melanoma. **(A)** The relative expression of RSK2 mRNA in normal and melanoma tissues was analyzed by GEPIA2 database. **(B)** The relative expression of RSK2 mRNA in different subtypes of melanoma and normal skin tissues was analyzed by GEPIA2 database. **(C)** The mRNA levels of RSK2 in normal and melanoma tissues were analyzed by GEO dataset (GSE46517). **(D)** The protein levels of RSK2 in normal and melanoma tissues were analyzed by the Human Protein Altas databases. **(E)** The prognostic value of RSK2 in melanoma patients in the overall survival was analyzed by GEO dataset (GSE22155). **(F)** The association of RSK2 mRNA expression with the clinical stage of melanoma was analyzed by GEPIA2 database. **(G)** The association of RSK2 mRNA expression with the age of melanoma patients in TCGA. Values represented as mean ± SD. ∗*p* < 0.05, ∗∗*p* < 0.01 vs. the Con group.

To investigate the role of RSK2 in melanoma, we first analyzed the mRNA expression of RSK2 between melanoma and normal skin tissue using the GEPIA2 (http://gepia2.cancer-pku.cn/). The results indicate that the mRNA expression of RSK2 was significantly higher in melanoma samples than that in normal tissue (Figure 1A). We further analyzed the mRNA expression of RSK2 in different subtypes of melanoma. As shown in Figure 1B, RSK2 was highly expressed in all subtypes of melanoma compared with normal skin tissue. Compared the mRNA expression of RSK2 in the GSE46517 dataset from the GEO database (https://www.ncbi.nlm.nih.gov/geo/), it is also shown that RSK2 was highly expressed in melanoma (Figure 1C). To investigate RSK2 protein expression in human melanoma and normal skin tissue, we analyzed the immunohistochemistry results using the Human Protein Atlas database (http://www.proteinatlas.org/). We found that RSK2 protein was highly expressed in melanoma tissue but not detected in normal skin tissue (Figure 1D). Importantly, we found that the high expression of RSK2 in melanoma patients was associated with poor overall survival (OS) (Figure 1E). In addition, RSK2 mRNA levels were higher in the advanced-stage melanoma relative to the early-stage ones (Figure 1F). RSK2 expression also showed a decreasing trend with the age of melanoma patients (Figure 1G). Thus, these results indicate that RSK2 expression is increased in melanoma and may be a potential biomarker for predicting the prognosis of melanoma patients.

### RSK2 promotes melanoma cell proliferation

**FIGURE 2:**
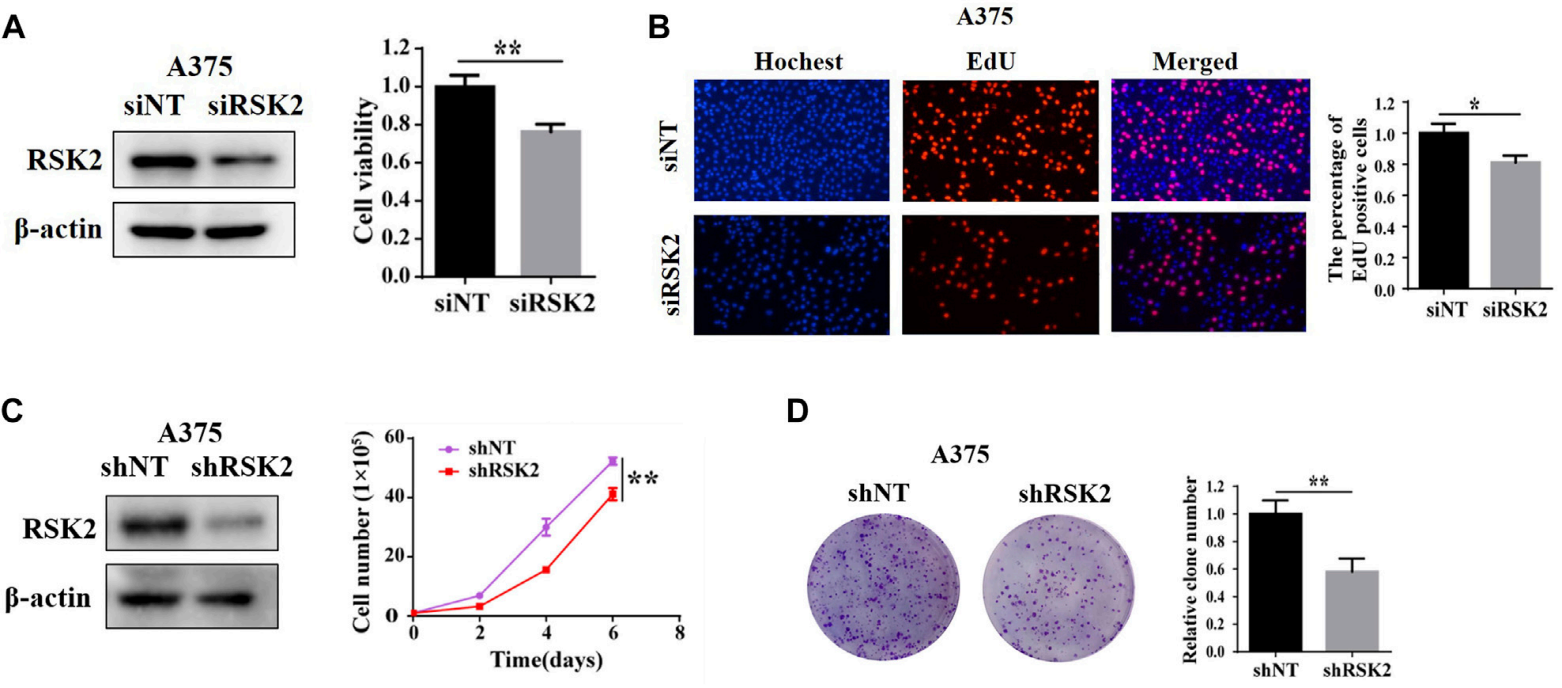
RSK2 promotes melanoma cell proliferation. **(A)** A375 cells were transfected with siNT and siRSK2, the expression of RSK2 protein was analyzed by Western blot and cell viability was measured using CCK-8 analysis. **(B)** The relative quantification of EdU-positive cells was exhibited. **(C)** A375 cells were transfected with shNT and shRSK2, the expression of RSK2 protein was analyzed by Western blot and the growth curve was determined. **(D)** The quantification of relative clone number was exhibited. ∗*p* < 0.05, ∗∗*p* < 0.01 vs. the Con group.

To further explore the functional role of RSK2 in melanoma cells, we knock-downed RSK2 expression in human melanoma A375 cells, and compared the cell proliferation in cells with or without RSK2 siRNA. As shown in Figure 2A, silencing of RSK2 decreased A375 cell viability as evidenced by CCK-8 assays. Silencing of RSK2 also dramatically decreased the percentage of EdU-positive A375 cells (Figure 2B). We also stably knock-downed RSK2 expression using lentiviral-mediated shRNA in A375 cells, and found that A375 cells transfected with RSK2 shRNA showed a significant reduction in cell number (Figure 2C) and colony formation (Figure 2D), compared with cells transfected with nontargeting shRNA. These results suggest that RSK2 promotes melanoma cell proliferation.

### Silencing of RSK2 increases vemurafenib sensitivity in melanoma

**FIGURE 3:**
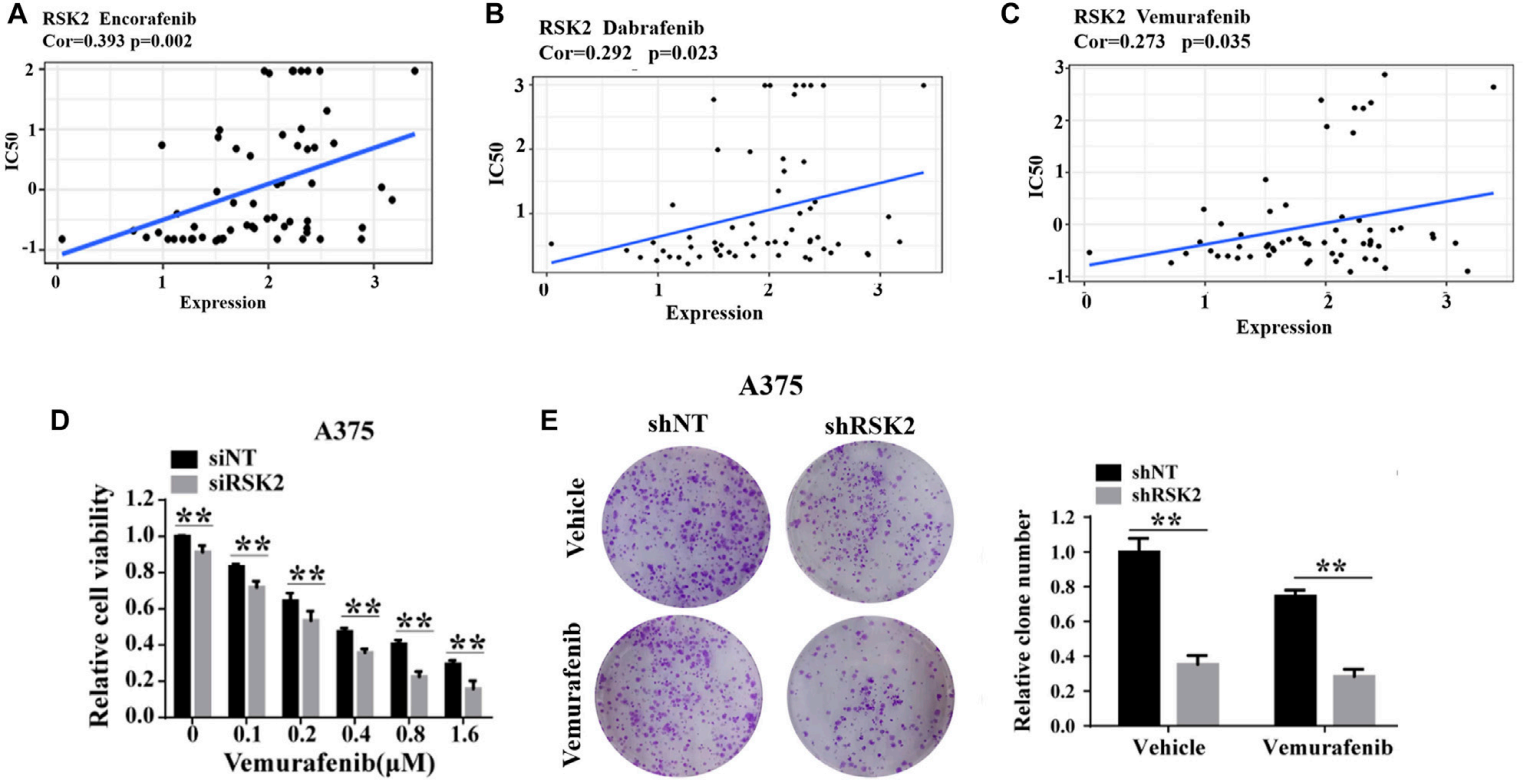
RSK2 is related with vemurafenib resistance in melanoma. The correlation between RSK2 expression and IC50 values of Encorafenib **(A)**, Dabrafenib **(B)** and Vemurafenib **(C)**. The Pearson’s correlation coefficient was calculated using R software. **(D)** The cell viability of A375 cells under increasing dose of vemurafenib treatment was assessed by CCK-8 assay. **(E)** The cell proliferation of shNT or shRSK2-transfected A375 cells with vemurafenib treatment was estimated via clonogenicity assay. Values represented as mean ± SD. ∗*p* < 0.05, ∗∗*p* < 0.01 vs. the Con group.

Resistance to vemurafenib is thought to be mediated by ERK1/2 activation in some melanoma cases. As a downstream kinase of ERK1/2, we further explored whether RSK2 is involved in vemurafenib resistance in melanoma. Firstly, we downloaded the IC50 values of anti-cancer drugs and gene expression profiles in the melenoma cell lines from the Cell Miner Analysis Tool project (http://discover.nci.nih.gov/cellminer/). The IC50 values of Encorafenib, Dabrafenib and Vemurafenib were positively correlated with RSK2 expression (Figures 3A–C), indicating that patients exhibiting high RSK2 expression may be resistant to BRAF inhibitor treatment. Next, we demonstrated that silencing RSK2 increased the sensitivity of melanoma cells to vemurafenib, as evidenced by CCK-8 and colony formation (Figures 3D,E). These results suggest that silencing RSK2 increased the sensitivity of melanoma cells to vemurafenib.

### RSK2 is associated with melanoma cell vemurafenib resistance

**FIGURE 4:**
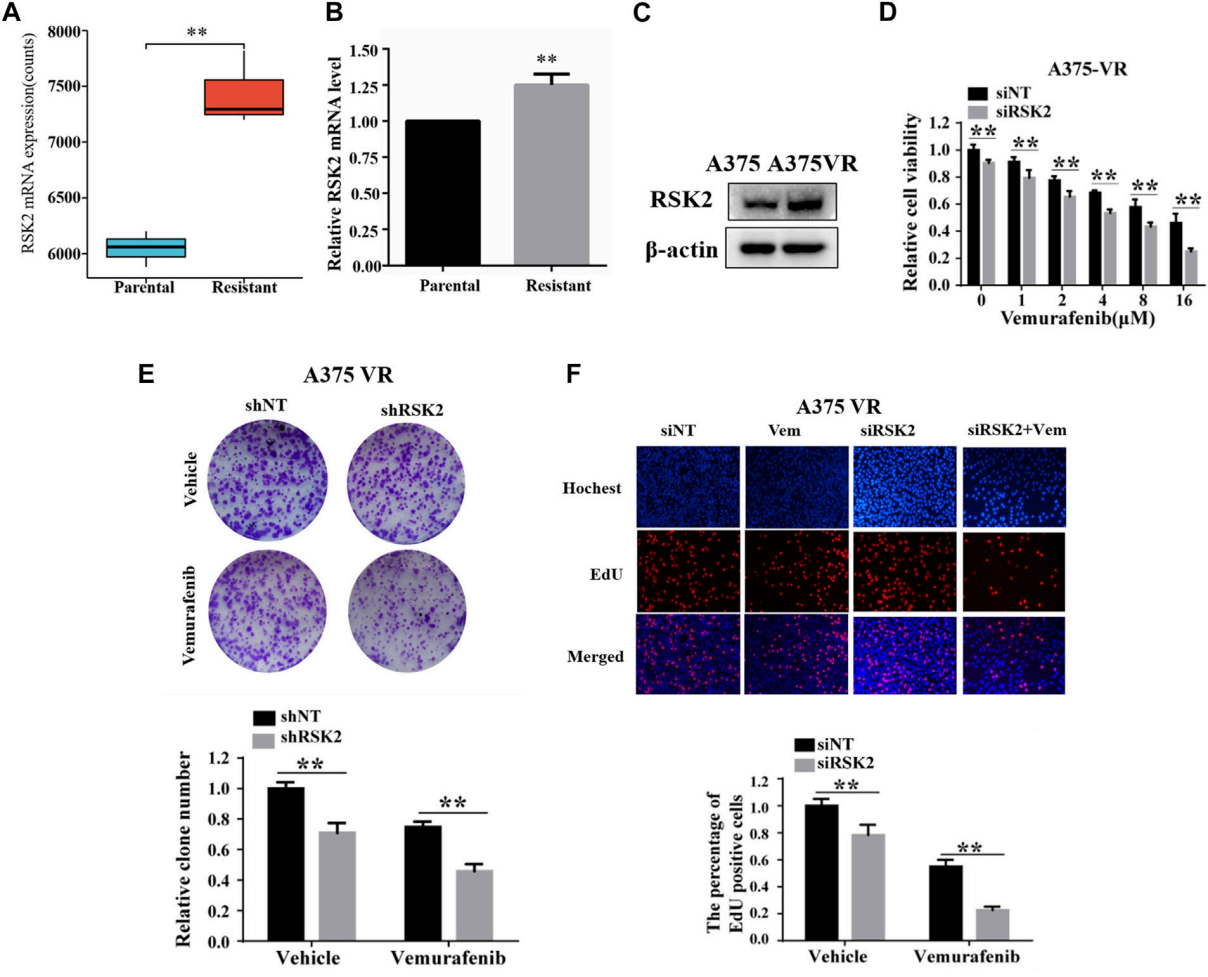
RSK2 promotes melanoma cell vemurafenib resistances. **(A)** RSK2 mRNA expression levels in A375 and A375-VR cells (GSE99923). **(B)** RT-qPCR was used to measure RSK2 mRNA expression in A375 parental cells and A375 vemurafenib resistant cells. **(C)** The protein levels of RSK2 were detected in A375 or A375-VR cells by Western blot. **(D)** Cell viability of siNT or siRSK2-transfected A375-VR cells under increasing dose of vemurafenib treatment was assessed by CCK-8 assay. **(E)** The cell proliferation of shNT or shRSK2-transfected A375-VR cells with vemurafenib treatment was estimated via clonogenicity assay. **(F)** EdU staining of indicated cells. Values represented as mean ± SD. ∗*p* < 0.05, ∗∗*p* < 0.01 vs. the Con group.

We further analyzed the role of RSK2 in vemurafenib resistance. Firstly, we found that the mRNA expression of RSK2 was significantly increased in A735-VR cells compared to the sensitive group in the GSE99923 dataset (Figure 4A). The mRNA level of RSK2 was further demonstrated higher in vemurafenib resistance A735-VR cells than that in A375 cells (Figure 4B). Furthermore, we found that the expressions of RSK2 protein were upregulated in A735-VR cells as compared to A375 cells (Figure 4C). We further investigated the potential role of RSK2 in vemurafenib resistant cells. Silencing the expression of RSK2 could increase the sensitivity of A375-VR cells to vemurafenib (Figures 4D,F). Together, these results suggested that RSK2 promotes the vemurafenib resistance in A375-VR cell line.

### RSK2 induces vemurafenib resistance through upregulating cyclin D1 expression

**FIGURE 5:**
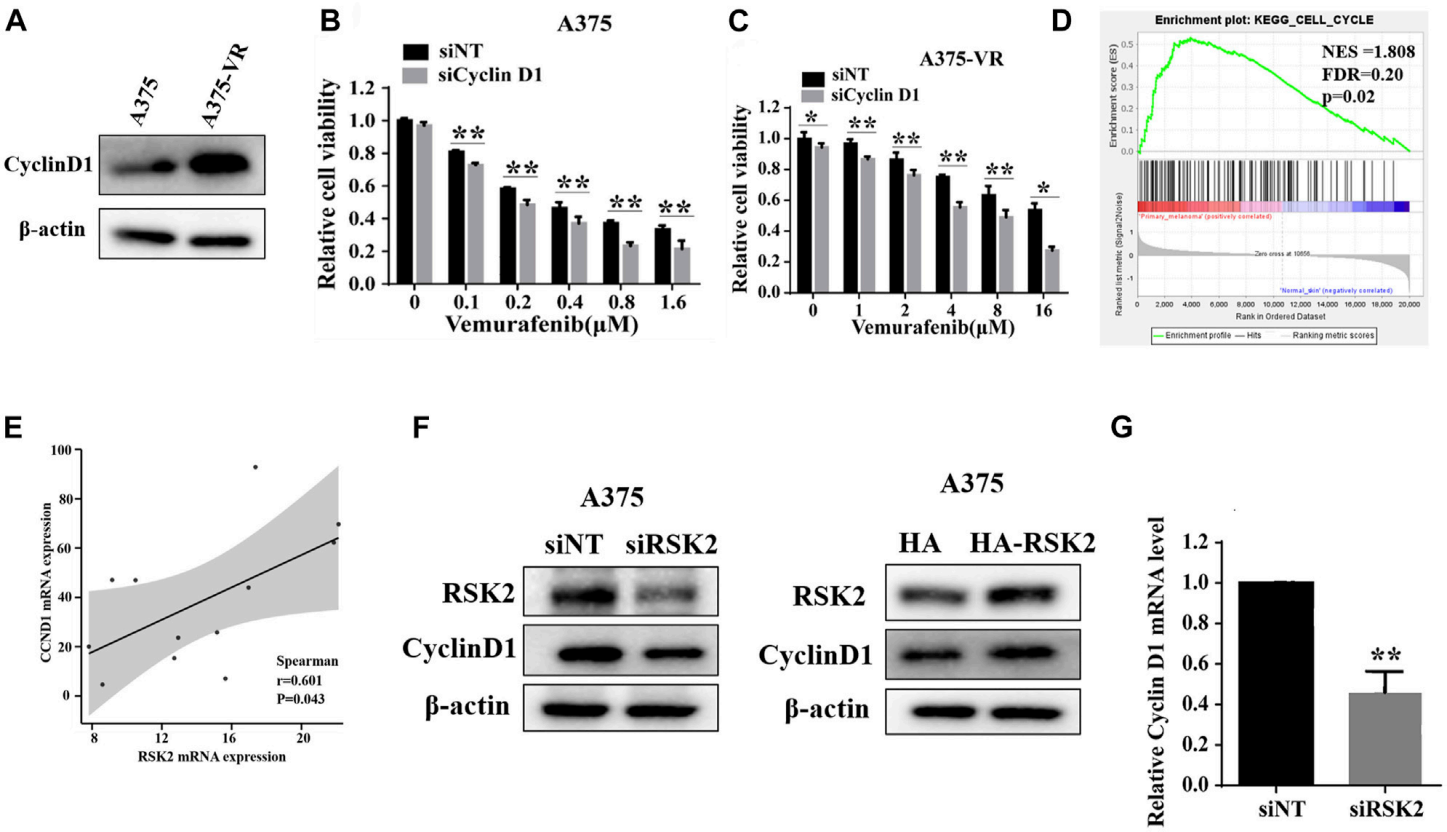
RSK2 induces vemurafenib resistance through upregulating cyclin D1 expression. **(A)** The protein levels of cyclin D1 were detected in A375 and A375-VR cells by Western blot. A375 cells **(B)** and A375-VR cells **(C)** were transfected with siNT or si CyclinD1, cell viability was assessed by CCK-8 assay. **(D)** Enrichment plots from gene set enrichment analysis (GSEA) of RSK2 in cell cycle by GEO dataset GSE15605. **(E)** The correlation between RSK2 and CCND1 predicted by GEO dataset GSE77940. **(F)** Western blot was used to measure cyclin D1 expression in A375 cells transfected with RSK2 siRNA or HA-RSK2 plasmid. **(G)** RT-qPCR was used to measure cyclin D1 expression in A375 cells transfected with RSK2 siRNA. Values represented as mean ± SD. ∗∗*p* < 0.01 vs. the Con group.

It has been reported that cyclin D1 elevation is associated with vemurafenib resistance. We found that the expression of cyclin D1 is indeed increased in vemurafenib-resistant A375-VR cells as reported (Figure 5A). Depletion of cyclin D1 increases the sensitivity of vemurafenib both in the vemurafenib-sensitive A375 cells and vemurafenib-resistant A375-VR cells (Figures 5B,C). To explore whether there is a regulatory relationship between RSK2 and cyclin D1, GSEA analysis shows that RSK2 is significantly enriched in the cell cycle signaling (Figure 5D). RSK2 is positively associated with CCND1 in mRNA level in a GSE77940 dataset (Figure 5E). We further transfected RSK2 siRNA and overexpression plasmid into the A375 cells, and measured the expression of cyclin D1. As shown in Figure 5F, the protein expression of cyclin D1 was decreased or increased in cells transfected with RSK2 siRNA or RSK2 plasmid. Furthermore, the mRNA level of cyclin D1 was also decreased by RSK2 knockdown (Figure 5G), indicating that RSK2 may upregulate cyclin D1 at the transcriptional level. These results indicated that RSK2 confers vemurafenib resistance in melanoma cells through regulating cyclin D1.

### RSK2 upregulates cyclin D1 *via* promoting FOXO1 degradation

**FIGURE 6:**
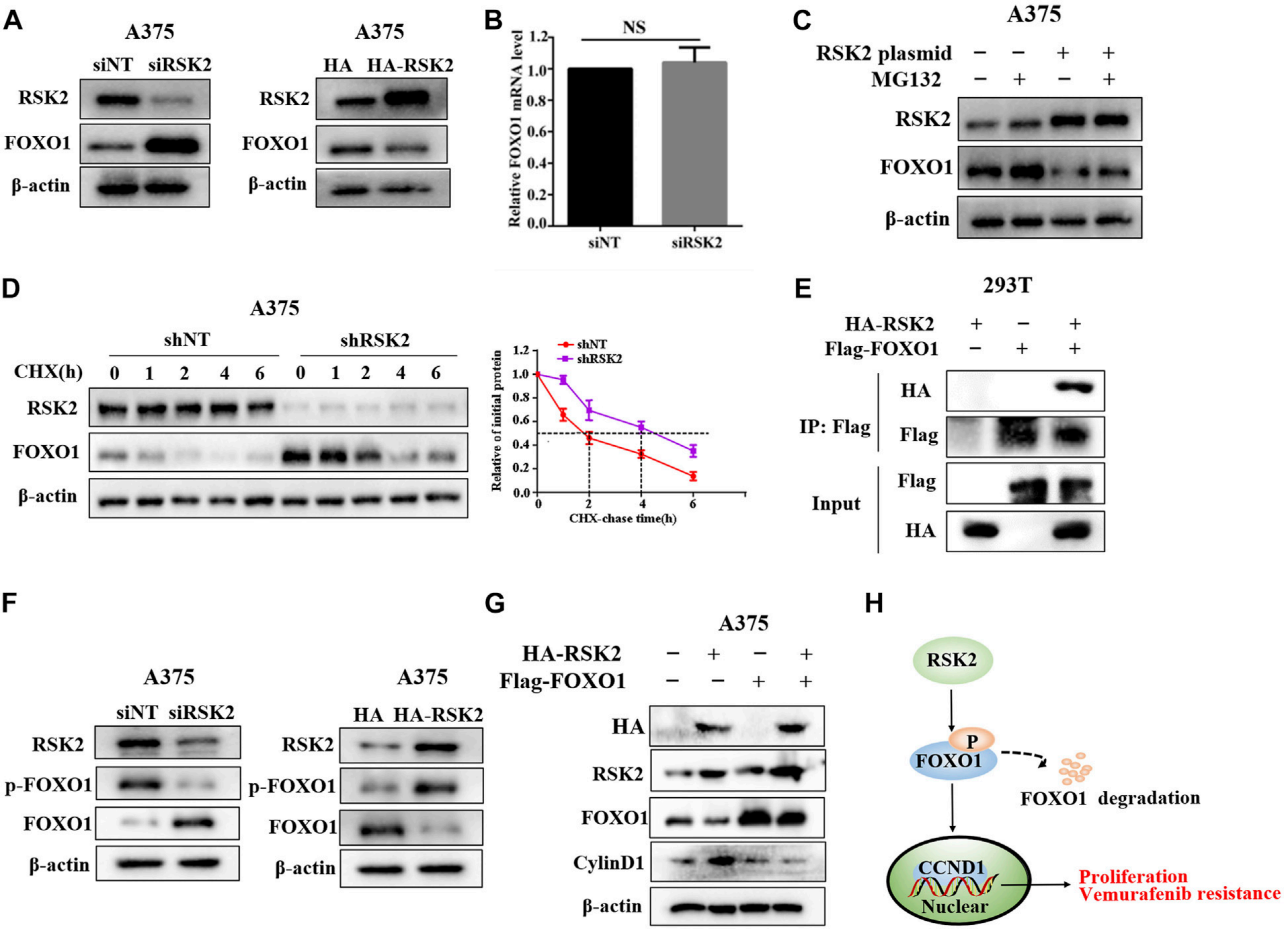
RSK2 upregulates cyclin D1 via promoting FOXO1 degradation. **(A)** Western blot was used to measure FOXO1 expression in A375 cells transfected with RSK2 siRNA or HA-RSK2 plasmid. **(B)** RT-qPCR was used to measure FOXO1 mRNA expression in A375 cells transfected with RSK2 siRNA. **(C)** The influence of RSK2 on FOXO1 degradation was estimated in A375 cells under MG132 treatment. **(D)** The effect of RSK2 on FOXO1 protein stability was evaluated in A375 cells under CHX treatment for the indicated time. **(E)** HEK293T cells were co-transfected with Flag-FOXO1 and HA-RSK2 plasmid as indicated, immunoprecipitation with anti-Flag antibody was performed. **(F)** Western blot was used to measure p-FOXO1 (S319) and FOXO1 expression in A375 cells transfected with RSK2 siRNA or HA-RSK2 plasmid. **(G)** Western blot was used to measure cyclin D1 expression in A375 cells co-transfected with Flag-FOXO1 and HA-RSK2 as indicated. **(H)** A schematic model of RSK2–FOXO1–cyclin D1 axis leading to melanoma proliferation and vemurafenib resistance.

As the transcription factor, FOXO1 has been reported to inhibit the expression of cyclin D1 (Wu et al., 2019). We next investigated whether FOXO1 is involved in the regulation of cyclinD1 by RSK2. We found that RSK2 knockdown increased the protein expression of FOXO1, while RSK2 overexpression inhibited FOXO1 expression (Figure 6A). Depletion of RSK2 did not affect the mRNA level of FOXO1 (Figure 6B). We next investigated how RSK2 regulates FOXO1 expression in melanoma cells. The reduction of FOXO1 regulated by RSK2 overexpression was inhibited by the proteasomal inhibitor MG132, indicating that RSK2 decrease FOXO1 by promoting its proteasomal degradation (Figure 6C). In addition, depletion of RSK2 markedly enhanced the half-life of FOXO1 under CHX treatment (Figure 6D). A co-immunoprecipitation (Co-IP) experiment demonstrated that RSK2 interacted with FOXO1 (Figure 6E). The phosphorylation of FOXO1 at T24, S256, and S319 can promote FOXO1 degradation (Zhu et al., 2015). We found that silencing RSK2 decreased FOXO1 phosphorylation at Ser319, while overexpression of RSK2 increased FOXO1 phosphorylation at Ser319 (Figure 6F), suggesting that RSK2 promotes FOXO1 degradation by regulating its phosphorylation. Furthermore, the upregulation of cyclin D1 by RSK2 overexpression was inhibited by FOXO1 overexpression, indicating that FOXO1 mediates RSK2-induced increased expression of cyclin D1 (Figure 6G). Together, these results indicated that RSK2 increases cyclin D1 by promoting FOXO1 degradation in melanoma cells.

## Discussion

In recent years, many studies have demonstrated that RSK2 plays a central role in the cell proliferation, metastasis and survival in various tumors and targeting it may improve the clinical efficiency of cancer therapies (Kang and Chen, 2011; Ma et al., 2018; Yoo et al., 2019). However, the molecular mechanism of RSK2 in vemurafenib resistance is not very clear. In this study, we found that RSK2 is highly expressed in melanoma tissue compared to normal skin tissue, and silencing of RSK2 inhibits melanoma cell proliferation. Furthermore, there is more expression of RSK2 in vemurafenib-resistant melanoma cells compare with vemurafenib-sensitive cells, and silencing the expression of RSK2 could increase the sensitivity of A375-VR cells to vemurafenib. Our results reveal that RSK2 not only promotes melanoma cell proliferation, but also mediates vemurafenib resistance through the upregulation of cyclin D1 mediated by accelerating the degradation of FOXO1 by phosphorylation (Figure 6H).

Previous studies demonstrated that RSK2 is a pivotal kinase to regulate human skin cancer cell proliferation and growth by promoting the phosphorylation of LKB1 or the activation of mTORC1 in melanoma (Zheng et al., 2009; Romeo et al., 2013; Zhang et al., 2019). However, the role and regulatory mechanism of RSK2 on melanoma cell proliferation remains to be further explored. Our present results provided concrete evidences for the role of RSK2 in melanoma cell proliferation by ectopic overexpression and knockdown of RSK2. We also proved that RSK2 expression was higher in melanoma tissue compared with the normal tissue and its overexpression was associated with increased pathological stage in melanoma patients. Prior study reported that RSK2 located in nucleus could enhance breast cancer cell proliferation through upregulation of cyclin D1 mRNA and protein level (Eisinger-Mathason et al., 2008). A natural anticancer agent silybin was found to induce cell cycle arrest and attenuate melanoma cell growth. The mechanism might suppress the expression of a cell cycle regulatory protein cyclin D1 by blockading the kinase activity of MEK 1/2 and RSK2 (Lee et al., 2013). Here, we confirmed the mechanism of RSK2 in promoting melanoma cell proliferation was to elevate cyclin D1 transcription. Transcription factor FOXO1 has been reported to be involved in the transcription of cyclin D1 (Sulzmaier and Ramos, 2013; Yang et al., 2019). Depletion of FOXO1 could promote cancer cell proliferation by enhancing cyclin D1 expression (Mao et al., 2015). In this work, we found that knockdown of RSK2 in melanoma cells did not affect FOXO1 mRNA expression but increased its protein level. We further revealed that inhibition of RSK2 improved FOXO1 protein expression by preventing its degradation. Phosphorylation at three conserved residues S319, S256, T24 by various protein kinases results in the translocation of the FOXO protein from the nucleus to the cytoplasm, thereby promoting its degradation by the 26S proteasome (Yamagata et al., 2008). In this study, we found that RSK2 interfered with FOXO1 and enhanced FOXO1 phosphorylation at S319, suggesting the mechanism of FOXO1 degradation mediated by RSK2 is due to phosphorylation. We also verified that overexpression of FOXO1 abrogated the increase of cyclin D1 induced by RSK2 overexpression. Taken together, our results revealed that FOXO1 is a novel RSK2 substrate and discovered a new RSK2–FOXO1–cyclin D1 cascade involved in melanoma cell proliferation.

The most common mechanism for BRAF inhibitors resistance is MAPK pathway reactivation in melanoma (Hatzivassiliou et al., 2010; Nazarian et al., 2010). Selective BRAF inhibitor Dabrafenib combined with a selective MAPK kinase (MEK) inhibitor trametinib successfully prolonged the progression-free survival of melanoma patients with BRAF^V600E^ mutations in a phase I/II clinical trials (Flaherty et al., 2012). However, this effect lasted only for an average of 9.4 months, drug resistance appeared again (Wagle et al., 2014). Recent study has shown that RSK inhibitors BI-D1870 and BRD7389 significantly reduced the proliferation of BRAF mutant melanoma cells that have acquired resistance to dual BRAF and MEK inhibitor treatment (Theodosakis et al., 2017). Researchers also found that inhibition of RSK2 by CF-X9, a RSK2 inhibitor discovered by virtual screening, hindered the cell growth in BRAF inhibitor-resistant melanoma cells (Zhang et al., 2019). Consistent with these reports, we found that RSK2 silencing not only improved the sensitivity of A375 cells to vemurafenib, but also overcame vemurafenib resistance in A375-VR cells. Besides reactivating MAPK, PI3K/Akt and EGFR signaling have also been involved in BRAF inhibitor resistance in melanoma (Chi et al., 2014; Laurenzana et al., 2019).

In addition, glutamine dependence, autophagy and mitochondrial biogenesis have also been implicated in resistance to BRAF inhibitor therapy (Hernandez-Davies et al., 2015; Martin et al., 2017; Carpenter et al., 2019). Blocking a single pathway seems to be not sufficient for completely reversing drug resistance. It was reported that the increase of cyclin D1 and the reactivation of MAPK coexist in vemurafenib-resistant tumors (Yadav et al., 2014). Our study discovered that cyclin D1 expression was upregulated in vemurafenib-resistant melanoma cells, accompanied by the increase of RSK2. Inhibition of cyclin D1 restored the sensitivity of the vemurafenib-resistant melanoma cells to vemurafenib mediated by RSK2. Together, we have uncovered a new mechanism RSK2-mediated phosphorylation and stabilization of FOXO1, thereby increasing cyclin D1 expression, is involved in promotion of melanoma cell proliferation. Inhibition of RSK2 can enhance sensitivity of BRAF mutant melanoma cells to vemurafenib and overcome vemurafenib resistance. These findings not only supported RSK2 as a critical oncoprotein in supporting cancer cell proliferation, but also provide a new mechanism by which RSK2 confers vemurafenib resistance in melanoma, suggesting that targeting RSK2 may be a potential strategy for melanoma treatment.

## Data Availability

The raw data supporting the conclusion of this article will be made available bythe authors, without undue reservation.
